# ‘Toning’ up hypotonia assessment: A proposal and critique

**DOI:** 10.4102/ajod.v5i1.231

**Published:** 2016-05-26

**Authors:** Pragashnie Govender, Robin W.E. Joubert

**Affiliations:** 1 Discipline of Occupational Therapy, School of Health Sciences, University of KwaZulu-Natal, South Africa

## Abstract

**Background:**

Clinical assessment of hypotonia is challenging due to the subjective nature of the initial clinical evaluation. This poses dilemmas for practitioners in gaining accuracy, given that the presentation of hypotonia can be either a non-threatening or malevolent sign. The research question posed was how clinical assessment can be improved, given the current contentions expressed in the scientific literature.

**Objectives:**

This paper describes the development and critique of a clinical algorithm to aid the assessment of hypotonia.

**Methods:**

An initial exploratory sequential phase, consisting of a systematic review, a survey amongst clinicians and a Delphi process, assisted in the development of the algorithm, which is presented within the framework of the International Classification of Functioning, Disability and Health. The ensuing critique followed a qualitative emergent–systematic focus group design with a purposive sample of 59 clinicians. Data were analysed using semantical content analysis and are presented thematically with analytical considerations.

**Results:**

This study culminated in the development of an evidence-based clinical algorithm for practice. The qualitative critique of the algorithm considered aspects such as inadequacies, misconceptions and omissions; strengths; clinical use; resource implications; and recommendations.

**Conclusions:**

The first prototype and critique of a clinical algorithm to assist the clinical assessment of hypotonia in children has been described. Barriers highlighted include aspects related to knowledge gaps of clinicians, issues around user-friendliness and formatting concerns. Strengths identified by the critique included aspects related to the evidence-based nature of the criteria within the algorithm, the suitability of the algorithm in being merged or extending current practice, the potential of the algorithm in aiding more accurate decision-making, the suitability of the algorithm across age groups and the logical flow. These findings provide a starting point towards ascertaining the clinical utility of the algorithm as an essential step towards evidence-based praxis.

## Introduction

Accurate assessment for early detection and appropriate intervention remains a priority for all clinicians involved in the field of paediatrics. Over the last decade the clinical assessment of hypotonia has been problematic, primarily due to the subjective nature of clinical evaluation. This has posed dilemmas for practitioners in reaching consensus on the veracity of the clinical assessment, given that the presentation of hypotonia in paediatric neurological morbidities can be either a benign or malignant sign (Bodensteiner 2008; Leyenaar, Camfield & Camfield 2005). This study was thus undertaken in a move towards advocating for more accurate and informed decision-making during clinical assessment of children with hypotonia.

The term *hypotonia* is used to describe low or decreased muscle tone. Hypotonia is a symptom of a number of neurodevelopmental, neurological and genetic disorders. Clinical evaluation of hypotonia is one of the aspects of the diagnostic and therapeutic process that remains subjectively assessed and thus creates an accuracy predicament for practitioners (Martin *et al*. 2005, 2007). There are currently no standardised assessment tools for children (after infancy and during early childhood) with low muscle tone (Soucy *et al*. 2015). Moreover, the incidence of hypotonia is difficult to ascertain given the fact that it is a symptom of a number of conditions or disorders (Lisi & Cohn 2011).

## Clinical assessment for early detection

Assessing disorders of muscle tone is essential in the clinical characterisation of children with numerous neurological disorders (Soucy *et al*. 2015). There are however a number of underlying causes of hypotonia for which there are no definitive laboratory or imaging tests, such as idiopathic hypotonia, so the role of clinical and developmental assessments remains important (Harris 2008). To date, there have not been any studies that have investigated non-invasive, objective clinical measures and the presence of a clinical diagnosis of hypotonia (Soucy *et al*. 2015).

The authors became increasingly concerned about the variability of decisions made in the assessment of hypotonia, the nature of the process and outcomes, the inaccuracy of which often contribute to delayed diagnosis and delayed interventions (Maulik & Darmstad 2007) as seen in the South African context. The World Health Organization (WHO & UNICEF 2012) advocates for a comprehensive approach towards appropriate paediatric care and support, including early identification; assessment and early intervention planning; provision of services; and monitoring and evaluation. In this study, the authors respond to the call from the WHO in ensuring early identification and intervention.

In an attempt to guide the assessment practices of clinicians that are responsible for the evaluation of low muscle tone in children, the development of a guided decision-making process was considered in the form of a clinical algorithm. A clinical algorithm is a text format that is especially suited for representing a sequence of clinical decisions for guiding patient care (Margolis 1983); it is said to be ‘a widespread instrument for increasing efficacy and managing quality in medicine by the implementation of specified standards into a systematic, logical, evidence-based, and rational concept’ (Khalil *et al*. 2011). There is evidence that the use of algorithms can assist in standardising care and in assisting effective diagnostic interventions (Miller, Delgado & Iannaccone 1993). The aim of this paper is to introduce the first prototype of the clinical algorithm and to present an initial critique that represents the voices of clinicians for whom this instrument has been developed.

## Materials and methods

The study methods are presented in two parts, namely, the development of the clinical algorithm and the critique of the algorithm for practice.

### Development of the algorithm

#### Choosing the best option

A process was initiated to determine whether a step-by-step scheme, decision tree or algorithm was most suitable for development, based on the question that was to be answered by this study. These concepts are often used synonymously, although they are said to represent different types of formal instructions for handling a particular subject (Khalil *et al*. 2011). Step-by-step schemes, decision trees and algorithms are described by characteristics according to *problem orientation, priority orientation, branching, loops, linear structure* and in having *several end points* (Khalil *et al*. 2011:32). An *algorithm* is said to fulfil all of these characteristics and was hence the primary choice. Application of recommendations by these authors (Khalil *et al*. 2011) was considered in the planning and development of the clinical algorithm.

#### Preliminary data and process of development

As this study was part of an ongoing larger project, the data from initial stages (Naidoo 2013a, 2013b; Naidoo & Joubert 2013) assisted in framing the stance that was to be taken in the development of the algorithm. The details of these phases are described in the respective papers.

The principal author drew on findings from the literature review, by identifying a candidate set of tests and methods that were used by clinicians. These were then proposed to a national cohort of clinicians (occupational therapists, physiotherapists and paediatricians), who indicated those tests and methods that are currently used in practice. These items were then exposed to a Delphi process, which aided in the reduction of items based on a consensus process. Twenty-four clinical characteristics, organised into 11 clusters, were determined as relevant for inclusion. For each characteristic, one test (as a first-line assessment method) had been identified. Using a desktop approach, the principal author used logical speculation from this data set with a level of abductive reasoning (an inference to the best explanation from logical deduction) to formulate and format the clinical algorithm in line with the technical regulations proposed by the International Organization for Standardization (ISO) norm (Khalil *et al*. 2011). The International Classification of Functioning, Disability and Health (ICF) multipurpose classification was also considered in the quest towards use of universal terminology. A process of reflection-on-action (Schön 1983) was also used in this developmental process, which included reflection on prior data collection processes, theoretical perspectives gained and consideration of knowledge gained. These combined culminated in the first prototype of the clinical algorithm, which is presented on one page with a logical flow, left to right, with adherence to the symbols and norms as required for clinical algorithms with aspects that were evidence-based from preceding phases.

## Critique of the algorithm

### Design

**FIGURE 1:**
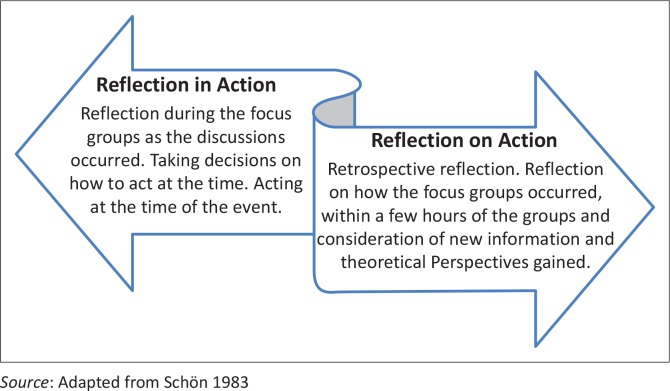
Processes of reflection-in-action and reflection-on-action in this study.

This aspect of the study followed a qualitative, emergent–systematic focus group design (Onwuegbuzie *et al*. 2009) in which focus groups were used for both exploratory and verification purposes. Kinsella (2010:567) noted that different disciplinary communities often work in silos, dealing with similar problems but not readily sharing knowledge around discipline lines, and asserted the place of reflection in the use and generation of knowledge for practice. The principal author thus considered the use of processes of reflection-in-action and reflection-on-action (Schön 1983) during this phase of the study (Figure 1), together with multiple iterations of focus group discussions.

### Sample and recruitment

Participation was invited via email correspondence in which the purpose of the study was explained. Practitioners working at district, regional and tertiary-level hospitals, private practice and schools were approached for participation. Purposive homogenous sampling was used to recruit participants. The final sample comprised 59 paediatric clinicians (i.e. paediatric occupational therapists, paediatric physiotherapists, paediatricians and paediatric neurologists). Paediatric clinicians were those practitioners who had experience with and were currently working with children with neurological disorders on a weekly basis. Additional selection criteria included a current registration with the Health Professions Council of South Africa and greater than 2 years of experience. Participants were recruited at different geographical locations over a year via the following methods: a workshop session at a national conference, special interest groups, multidisciplinary team meetings, district-level meetings and institutional fora. Data gathering occurred over a period of 12 months at sites convenient to the group of clinicians. These included forum meetings, a national conference and at hospital departments. Composition of the groups was by geographic working teams. Each group, by default, contained a mix of both newly trained clinicians as well as more experienced clinicians.

### Data collection strategy

Ten focus group discussions were held, varying between five and eight participants in each group and lasting 90–120 minutes. For the larger groups, a facilitator was included to aid the process. Each individual within the group were presented with an A3 printout of the algorithm, with a 10-minute didactic input on the development of the algorithm. This was followed by a set of 10 open-ended questions related to the scope and purpose, applicability and clarity of presentation.

The multiple focus group discussions allowed for the assessment of data redundancy in general and across-group redundancy and/or saturation in particular (Onwuegbuzie *et al*. 2009). Sampling ceased when across-group redundancy was achieved.

### Data analysis

The focus group discussions were digitally recorded. Tape-based analysis of audio-recordings (Onwuegbuzie *et al*. 2009) was done with semantical content analysis (Stewart & Shamdasani 2015:126). The principal author’s reflections-on-actions were also recorded as analytical memos during the data analysis process, which occurred within a maximum of 1 day following the data collection, and summarised.

Within semantical content analysis, the frequency of specific concepts (indicated in Table 1) that were mentioned (designation analysis) as well as phrases or descriptors that were used in the critique (attribution analysis) were noted. Additionally, some of the more specific analytical aspects, namely constant comparisons between groups, use of the group dynamics as a resource and use of participants as co-analysts (Barbour 2007; Kamberelis & Dimitriadis 2011), were considered. These items are outlined in Table 1.

**TABLE 1:** Analytical aspects considered in data analysis.

Analytical aspects	Application in study (critique)	Observations from the study (critique)
Constant comparisons Inter- and intra-group differences	Data were analysed after each focus group with analytical notes on the researcher’s reflection-on-action. The systematic application of constant comparison between individual voices and collective group’s voices in each of the focus groups discussions were noted and reflected upon.	A number of similarities occurred with therapists in homogenous groups. However, in the groups that included therapists, paediatricians and paediatric neurologists, although considered homogenous in this study, a number of differences were noted, with the emphasis in the assessment processes differing as expected.
Negotiating similarities and differences between groups	Similarities between groups were noted. Attention was given to similarities as implications were considered for the algorithm; data were interrogated to offer additional explanation and not just gloss over items.	Surprises in some groups were interrogated as part of the reflection-in-action process with respondent validation so that clarity was achieved.
Using group dynamics as a resource in analysis	The multidisciplinary groups assisted in interesting debate that also served as a resource in determining differing viewpoints and how these were negotiated in a team.	The synergy and dynamism generated within this homogenous collective revealed normative unarticulated norms and normative assumptions.
Focus group participants as co-analysts	Member-checking and verification was done simultaneously in order to understand viewpoints.	This respondent validation aspect was useful in ensuring that the feedback that was captured was accurate and allowed the participants to elaborate and clarify where necessary, thus adding to knowledge exchange within the sessions.
Personal and professional backgrounds as resources	Maximum variation sampling allowed for some diversity in the groups and provided the platform for debate across professions and disciplines.	Having groups of differently trained individuals from three professional groups with different paediatric experiences added to the richness of the data and provided somewhat of a realistic clinical situation.

*Source*: Adapted from Barbour (2007); Kamberelis and Dimitriadis (2011)

### Ethical considerations

Ethical principles of autonomy, veracity, beneficence and scientific honesty were observed throughout this study. Autonomy was ensured by soliciting participants’ consent to participate in the study and by assurance of and respect for their right to withdraw from the study at any time without prejudice. Beneficence was ensured by the researcher–participant relationship, privacy and confidentiality were maintained where possible, and ethical clearance was granted by the Human and Social Science Ethics Committee of the University of KwaZulu-Natal. Ethical considerations with respect to data storage and management were also ensured by password-protected devices and access control filing systems. Veracity was maintained by authenticity in the presentation of findings and by upholding the principle of scientific honesty and integrity. The participatory nature of the focus group sessions was explained and participants were advised at the outset about ‘shared expectations and the nature of participation’ so that there were no misconceptions or misunderstandings about ownership. These points were affirmed in signed declaration forms.

### Trustworthiness

In order to ensure trustworthiness of the study, Lincoln and Guba’s (1985) evaluative criteria of credibility, dependability and confirmability were noted and strategies implemented. Triangulation of sources (literature and practitioners) and data (focus group data from multiple groups), together with theory (literature, ICF) triangulation, was ensured in this study. Respondent validation or member checking occurred concurrently within the focus group sessions to reduce misinterpretations and researcher bias. This was done in the form of paraphrasing as well as momentary summaries and invitation for comment. Reflexivity was observed throughout the planning and execution of this study. Personal and intellectual biases were highlighted in a statement of positionality. There was the conscious experiencing of the self as both inquirer and respondent, as researcher and learner (Denzin & Lincoln 2011:124). The principal author’s contribution to the research study can be seen as useful and positive rather than detrimental and her position as a paediatric therapist and biases were highlighted prior to the discussion sessions. Measures that were put in place to maintain a level of objectivity included initial bracketing to facilitate the discussion – an opening statement on occupying a hybrid position in the research (Jootun, McGhee & Marland 2009) as researcher and practitioner.

## Findings

### Presentation of findings

**FIGURE 2:**
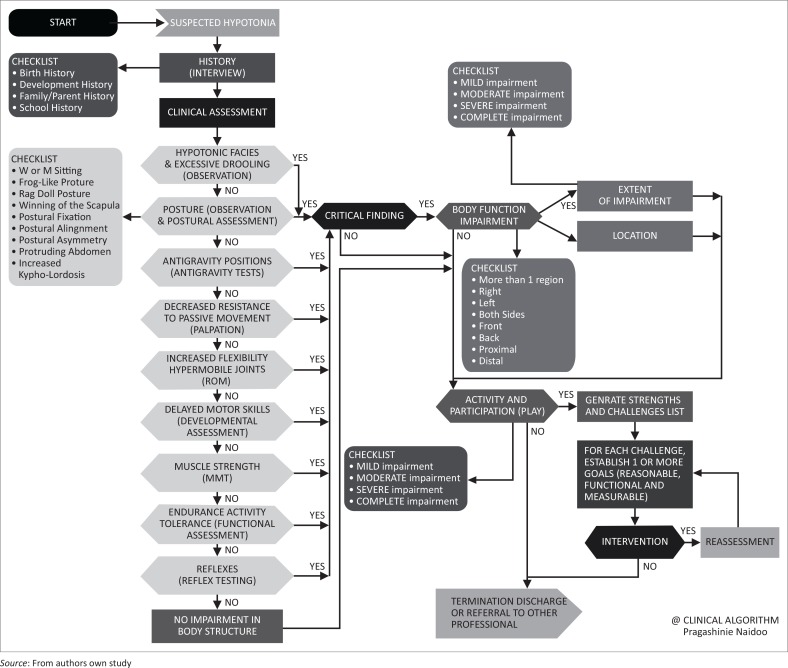
A clinical algorithm for hypotonia assessment.

This study resulted in the development of a clinical algorithm for paediatric clinicians in the assessment of hypotonia in children. This initial prototype is presented in Figure 2.

The second aspect of this study considered a critique of this algorithm with a purposive sample of 59 participants. Demographic data are presented in Table 2. The majority of participants (67%) were within the 30–49 year age range, with a high percentage of participants holding a qualification higher than that of a bachelor’s degree (*n* = 47%). The majority of the participants (*n* = 53%) were employed in a public hospital setting. The experience of participants ranged from newly qualified clinicians with less than 5 years of experience (17%) to greater than 20 years of experience (19%).

**TABLE 2:** Sample Demographics (*n* = 59).

Demographic Variables	Sample	

	*n*	*%*
**Age**		
20-29 years	13	22
30-39 years	25	42
40-49 years	15	25
50-59 years	3	5
> 59 years	3	5
**Highest qualification**		
Bachelor’s Degree	31	53
Postgraduate Diploma	3	5
Master’s Degree	23	39
Doctorate Degree	2	3
**Certifications**		
Neurodevelopment	13	22
Sensory Integration	6	10
Paediatric Neurology	8	14
NBAS	2	3
Griffiths	3	5
**Experience in the field of Paediatrics**		
< 5 years	10	17
5-10 years	15	25
11-15 years	13	22
16-20 years	10	17
> 20 years	11	19
**Practice Setting**		
School Based	2	3
Private Practice	4	7
Public Hospital	31	53
Academic	12	20
Combination of Settings	10	17
**Average Paediatric Case Load per week**		
< 5 paediatric cases per week	13	22
5-10 paediatric cases per week	13	22
11-15 paediatric cases per week	7	12
16-20 paediatric cases per week	5	8
> 20 paediatric cases per week	21	36

A description of the semantical content analysis (Stewart & Shamdasani 2015), that is, specific concepts that were mentioned in the focus groups (designation analysis), as well as phrases or descriptors that were used in this process of critique (attribution analysis), are presented in Table 3.

**TABLE 3:** Concepts and phrases from focus group analysis.

Description>	Concepts	Phrases/questions
Inadequacies Misconceptions Omissions	Developmental norms Hypotonia terminology	What is abnormal? Phasic versus postural tone? Hypotonia with/without weakness?
	ICF clarity	Extent and location? Inadequate knowledge of terms.
	Intervention	Intervention loops seem to be misleading.
	Quantification	Mild, moderate or severe descriptors? How many signs are needed for confirmation of hypotonia?
Strengths	Red flags	Is specific/pinpoints specifics/covers all bases. Highlights criteria and tests to use. Checklists are useful.
	Evidence-based	Based on research. Will aid decisions in assessment.
	Current practice	Can merge with and extend current practice.
	Logical flow	There is a process to follow.
	Age group	Can be used across ages.
	Structure	Structure for treatment plan is given.
	Problem solving	Assists problem solving in assessment.
Clinical use and enablers for implementation	Multidisciplinary team	Team approach and holistic assessment. Same terminology for all professional groups aids communication.
	Guidelines	Provision of guidelines will assist all levels of experience.
	ICF	ICF is culture-free and universal.
	Paper-based	Easy access for use.
Barriers to implementation and resource implications	Expertise	Is it part of a toolkit? Unfamiliarity with ICF.
	Time	It may be time-consuming.
	Multidisciplinary team	No interest in careful assessment in general practice settings.
	Training	Are clinicians to be trained in the use?
Appearance and flow	Language	What is a critical finding? Strengths and challenges?
	User-friendliness	Simpler terminology. Description of terms. Explanations for inexperienced clinicians.
	Flow	Difficult to follow logic and flow.
	Format	Arrows confusing. Make arrows bolder. Checklist boxes too close to main boxes.
	Size	Large version and a pocket version.
Recommendations	Use for other professions	Adapt to highlight red flags for nurses. Developmental red flags for interns.
	Education	Include in curriculums for target groups.

*Source*: From authors own study ICF, International Classification of Functioning, Disability and Health.

### Algorithm prototype with critique

The flow of the algorithm is sequentially presented as a narrative, interspersed with critical feedback from the focus group discussions. The visual representation of the algorithm appears in Figure 2.

The process begins on the far left at the symbol indicating *start.* The ‘message to network’ symbol indicates *suspected hypotonia*, as the diagnosis under decision. The first step in this process involves the collection of initial data and gaining a history. A one-way ‘process symbol’ indicates the mono-directional action of obtaining a *history* via an interview. A *checklist*, provided on the left, indicates those aspects that are deemed imperative as part of the history taking. These inclusions have been transposed from the Delphi process in the preliminary stage (Naidoo & Joubert 2013), in which panellists concurred with a high level of agreement that these aspects should be included. This is followed by another mono-directional action in which the *clinical assessment* is initiated. The hierarchy of inclusion of the clinical characteristic clusters that follow were based on the ranking orders gauged from the consensus process (Naidoo & Joubert 2013). One change was made to this hierarchy, in that *hypotonic facies* and *excessive drooling* were combined as the first characteristic under consideration and placed at the top of the hierarchy, given that these signs would be overt and noticeable to the clinician on first presentation at the clinical assessment. The characteristics that follow have been ranked in the order of most importance within the top five, top three and top two rankings from the consensus process.

Within each of the presented characteristics, the *preferred test and method*, which would now indicate the first line of assessment, are included in parenthesis. Posture and antigravity tests were ranked the highest and hence are presented higher up. A *checklist* for posture has been included on the left, as many characteristics fell within this cluster for consideration. The last characteristic listed is *reflexes.*

From the critique, clinicians appeared to appreciate the value of the red flags and the inclusion of evidence-based criteria and first line tests for use. There was also the suggestion of the algorithm assisting in decision-making and inclusion of criteria as a way of ‘covering all bases’ in assessment as well as a sense that these aspects of assessment could be merged and extend current assessment practices.

The algorithm proceeds with clinical characteristics, which are represented within decision symbols in which a binary decision (*yes* or *no*) has to be made. Each of the *no* responses lead to the next characteristic for consideration; if all responses are *no*, they lead directly to the next level of evaluation (*activity and participation*). The *yes* responses feed into the loop that indicates a finding, based on the patient’s developmental age, with a process box for the number of characteristics found to be problematic.

A persistent concern that arose from the critique was related to developmental norm expectations and at what point would symptoms be considered abnormal, as well as the number or the presence of criteria that would constitute an overall diagnosis of hypotonia. There were also debates around definitions of phasic and postural tone that required clarification and the presence of hypotonia with/without weakness and the implications thereof. In general, the critique sessions were useful in highlighting the possibility that less experienced clinicians may require additional guidelines for the algorithm to be of maximal benefit. Moreover, a number of clinicians appeared unfamiliar with terminology of the ICF, which indicated the need for potential training and knowledge translation.

Following the assessment of specific criteria and tests, the process flows to another process symbol, indicating *body structure impairment*. At this stage the clinician is able to then quantify the *extent of the impairment* and *location*, indicated as process boxes.

The critique revealed unfamiliarity with ICF terminology once more, as well as clinicians’ lack of awareness of descriptors for quantification (for example, mild, moderate, severe).

It is a concern that almost a decade and a half later clinicians remain unaware of this taxonomy, given that the ICF is the WHO’s international standard for measuring health and disability, in addition to being adopted by all 191 WHO member states in 2001 (WHO 2001).

There were a number of clinicians who however indicated that the inclusion of the ICF was beneficial given its universality.

The response from the process symbol, *body structure impairment (extent and location)*, as well as the loop from the *no* response on the clinical characteristics in determining low muscle tone then feeds into the *activity* and *participation impairment* decision symbol. This decision symbol has *play* within parenthesis, as this was indicated as being an important component from the preceding study (Naidoo & Joubert 2013). A *checklist* is once more provided for quantification of the impairment. The reason for ensuring that the loop returns to this stage is that a child requires holistic assessment and, if *body structure impairments* are not found, functional impairments still require investigation. The converse is also true, in that if there is *body structure impairment*, the child may inevitably have difficulties in functional areas that may be impacted by the *body structure impairment*.

From this point of the algorithm, the assessment components – *history* and *collateral* information, *body structure impairment, activity* and *participation –* are complete.

The aim of this study and of the development of this algorithm was to provide a guideline for decision-making. Ending the algorithm at this point would have negated the holistic management of the child. On the basis of the *body structure* and *activities and participation impairments,* a child’s *strengths and challenges* are generated and merged, and appropriate intervention goals are then developed. If intervention occurs, a reassessment loop is created and returns to *goal development*. The loop ends on the *output* symbol, which characterises *termination, discharge* or *referral to another professional*.

Therapists within the sample articulated the benefit of a therapeutic plan, including intervention for management of the child that is under assessment. This is in keeping with therapeutic goals of intervention which consider holistic care. Part of this holistic care includes referral to other professionals, which was indicated as being a crucial aspect to the team approach.

If intervention does not occur, it feeds into the loop that ends in *termination, discharge* and *referral to another professional.* The *no* loop from the *activity* and *participation* symbol also ends in the message from user (output symbol). *Multiple end points* and outputs may have been restrictive; hence all three were combined as opportunities for output.

## Discussion

The initial prototype of the clinical algorithm described in this paper contributes towards the holistic diagnosis of children who present with hypotonia. It was developed in response to the challenges expressed by the scientific community, given the value of the initial clinical assessment. The need for a common diagnostic language has been indicated in the literature for at least the last three decades (Coffin-Zadai 2007; Jette 1989; Norton 2007; Rose 1989; Sahrmann 1988) and continues into this decade (Martin, Westcott & Wrotniak 2013). Part of the need has been attributed to appropriate interventions and to assist with prognostication (Coffin-Zadai 2007; Jette 1989; Martin *et al*. 2013; Norton 2007; Rose 1989; Sahrmann 1988).

An evidence-based clinical algorithm was a suitable method of choice for ensuring a common language for decision-making in the assessment of hypotonia. Formulation of evidence-based algorithms is said to be an increasing practice in both scientific papers and textbooks; however, their usefulness has been questioned, as many of the authors have been found not to adhere to the formal requirements (Eitel, Kanz & Ma 2000; Khalil *et al*. 2011). The International Telecommunication Union norm symbols, based on the ISO norms, were incorporated more than a decade ago to adapt the algorithm for clinical practicability (Khalil *et al*. 2011) and have thus been adhered to in this study. The algorithm described has been developed to assist in guiding clinicians in following a systematic process (described in detail in the ‘Findings’ section) for identifying specific characteristics that are associated with low muscle tone, as well as in the choice of methods that will primarily aid the evaluation of the specific characteristics. It has been suggested that methods for writing clinical algorithms that represent expert consensus be sought in practice (Margolis 1983). Within this study, items included in the clinical algorithm, that is, clinical characteristics and methods and tests, have not been randomly assigned, but are rather the outcome of a previous rigorous consensus process (Naidoo & Joubert 2013). This clinical algorithm has also not been developed for implementation within any specific frame of reference, or within specific models of practice; however, aspects of the ICF have been superimposed, to assist quantification of impairments. The provision of a qualifier checklist thus allows the clinician to quantify the degree of impairment, which has previously been indicated as a dilemma (Naidoo 2013b). Moreover, the algorithm allows for holistic assessment by the inclusion of collateral information (history) and functional limitations (activity and participation), as well as by the provision of an end point following assessment – towards intervention or referral to another professional, all towards the goal of holistic management. This algorithm is thus in keeping with sensory integration, development, neurodevelopmental and biomechanical frames of reference, when applied to children with hypotonia, and may thus be useful in a number of paediatric settings with no resource implications.

Seeking agreement amongst clinicians should be seen as a starting point for establishing criteria that are likely to have significant clinical sensibility and that can be tested to ensure validity. As a result, the initial critique was deemed a necessary procedure towards determining the potential pitfalls and strengths prior to plans for implementation. A number of factors that may affect the clinical usefulness of the algorithm were highlighted. Barriers that were highlighted included aspects related to knowledge gaps of clinicians, issues around user-friendliness and formatting concerns. Knowledge gaps were associated with the symptom of hypotonia itself, where there was a lack of understanding amongst some clinicians around the difference between phasic and postural tone, as well as in isolating the presence of hypotonia with or without weakness. Previous studies (Bodensteiner 2008; Gowda, Parr & Jayawant 2008; Igarashi 2004; Jan 2007; Prasad & Prasad 2003, 2011; Van Toorn 2004) have clarified these aspects satisfactorily. However, this finding has highlighted the need to have these concepts clarified as an adjunct to the algorithm, towards a process of knowledge translation, in order to ensure that knowledge gaps will not impede the assessment process. Additional gaps were evident in developmental norm expectations and familiarity with ICF terminology. Martin *et al*. (2013) introduced the term ‘hypotonia syndrome’, which they defined based on their two previous studies (Martin *et al*. 2005, 2007), and in their description have drawn on the gross motor milestones as an indicator of developmental norms. Harris (2008) also reiterated the use of developmental tests, and together these serve as examples of norms when assessing hypotonia. Terminology commensurate with that of the ICF was ensured in the algorithm in an attempt to ensure universality. Unfamiliarity with terms can be addressed by clarification and definition of terms used again as an adjunct to the algorithm. User-friendliness and formatting were highlighted as additional barriers. User acceptability is essential and is sometimes dependent on whether the user believes that the proposal is a valid construct and whether the criteria set is easy to use (First *et al*. 2004). However, a clinician’s perception of a valid construct may be dependent on a number of personal factors, such as familiarity with the scientific literature, clinical experience and even practice setting (First *et al*. 2004). In order to ensure clinical acceptability, clarification of terms, provision of additional information and formatting aspects are considered essential towards the revision of the algorithm to ensure user uptake.

Strengths identified by the critique included aspects related to the evidence-based nature of the criteria included within the algorithm, the suitability of the algorithm in being merged or extending current practice, the potential of the algorithm in aiding more accurate decision-making, the suitability of the algorithm across age groups and the logical flow. These highlighted aspects contribute to our understanding of therapists in developing a diagnostic language for human movement systems and as being different from those articulated by physicians, which are often based in cellular pathology (Martin *et al*. 2013). As a result, a more comprehensive evaluation, which includes signs (physical manifestations observed in the assessment) and symptoms (client reports) were interpreted as having been achieved by the development of this algorithm, with the identification of early warning signs or red flags to assist in early detection for early intervention. Inevitably acceptance of this diagnostic language is essential in ensuring clinical utility of the algorithm. Enablers for implementation included the attraction of a multidisciplinary team approach; guidelines to assist clinicians that may require additional knowledge; the use of universal terminology; and the fact that the algorithm is paper-based and dependent on the clinician’s prior skills and knowledge. The ease of user-friendliness is crucial in understanding user acceptability (First *et al*. 2004) and hence other practical issues, such as the length of time it takes to assess a particular criteria, as well as explanations of terms and so on, may need clarification in order to increase user uptake. The authors are also cognisant of the fact that, as clinical experience evolves, the opinions of experts may also change, together with their assessment and diagnostic practices. Flexibility may thus be necessary so that the criteria may be re-examined and revised at intervals (Graham, Regehr & Wright 2003) as new information and research is developed.

## Conclusion

In this paper, the process of development of an initial prototype of an evidence-based clinical algorithm is described, together with a process of scrutiny and critique. As with any new development, this study has been useful in identifying important aspects that are essential to revision of the algorithm for practice. Aspects that have emerged include positive feedback with respect to the algorithm’s applicability and acceptability for practice, in addition to aspects critical for revision, such as concept clarification and a few practical issues. Although this paper presents an initial critique, the algorithm continues to require peer review and critique, and thus the authors invite readers to engage with the current proposal, so that continued work towards refinement through feedback may be realised, prior to initiating a process of validation.
